# Anti-L1 antibody-bound HPV16 pseudovirus is degraded intracellularly via TRIM21/proteasomal pathway

**DOI:** 10.1186/s12985-022-01826-x

**Published:** 2022-05-26

**Authors:** Meiying Li, Jianmei Huang, Yi Zhu, Ziyi Huang, Guonan Zhang, Jianming Huang

**Affiliations:** 1 Department of Biochemistry and Molecular Biology, Sichuan Cancer Institute, Chengdu, 610041 People’s Republic of China; 2 Department of Gynecologic Oncology, Sichuan Cancer Hospital, School of Medicine, University of Electronic Science and Technology of China, Chengdu, 610041 People’s Republic of China; 3 Department of Ultrasound, Sichuan Cancer Hospital, Chengdu, 610041 People’s Republic of China; 4 Department of Bioinformatics, Basic Medical College of Chongqing Medical University, Chongqing, People’s Republic of China

**Keywords:** HPV16, Persistent infection, Cervical cancer, TRIM21, Anti-L1 antibody, Intracellular neutralization

## Abstract

**Background:**

Persistent HPV16 infection is the leading risk factor for developing cervical cancer. Anti-L1 antibodies against HPV16 produced in HPV16 infections play diverse roles in the clearance of virus infection and prevention of persistence. It has been implicated that the cervicovaginal squamous epithelial cells actually express TRIM21 and that some HPV16 particles could escape leaky endosomal compartment into the cytosol and that Fc receptor TRIM21 directly neutralize infection by targeting antibody-opsonized viruses for proteasomal degradation. We explored whether anti-L1 antibody opsonized HPV16 pseudovirus (PsV) entered into the cytosol could be neutralized by TRIM21-mediated activation of a proteasomal pathway to reduce the chance of persistent HPV16 infection.

**Methods:**

HPV16 PsV were generated and extracted in HEK 293FT cells co-transfected with pcDNA3.1-eGFP and p16sheLL plasmids according to the standard protocol. The HPV16 PsV with capsid protein L1 was characterized by fluorescence microscopy and western blot, and the HPV16 PsV titer and anti-L1-bound PsV entry efficiency were detected by flow cytometry. The expressions of transcription factors (TF) and cytokines elicited by the TRIM21-activated proteasomal pathway were confirmed by dual-luciferase reporter assay and RT-qPCR. The changes in HPV16 PsV load with or without inhibitors in the infected HEK 293FT cells were determinated by qPCR.

**Results:**

Simultaneous transfection with pcDNA3.1-eGFP and p16sheLL plasmids into the HEK 293FT cells resulted in the self-assembly of HPV16 PsV with capsid protein L1. Both HPV16 PsV and anti-L1-bound HPV16 PsV could infect HEK 293FT cells. Anti-L1-bound PsV up-regulated TRIM21 mediated-activation of proteasome and increased expressions of TF and cytokines in the infected cells where HPV16 PsV load reduced by ~ 1000-fold in the presence of anti-L1 antibody, but inhibition of proteasomal activity increased HPV16 PsV load.

**Conclusion:**

Our preliminary results indicate that anti-L1 antibody entered with HPV16 PsV into the cells could mediate degradation of HPV16 PsV by TRIM21-activated proteasomal pathway intracellularly, giving anti-capsid protein L1 antibody a role in host defense of persistent HPV16 infection.

## Introduction

Overall it is estimated that 5.2% of all cancers are attributable to high-risk human papillomavirus (hrHPV) [1–3]. HPV16 is the most frequently occurring high-risk type and presents in ~ 50% of cases in most epidemiological and experimental studies [4, 5]. Virtually all natural history studies show that genital HPV infections are prevalent in young sexually active women with a cumulative prevalence of 60–80% [6]. However, in most cases of hrHPV infection, the HPV16 virus can be cleared spontaneously within 8–16 months post-infection [7].

Generally, HPV-induced lesion regression is due to a cell-mediated immune response to early proteins. It has been illustrated that the CD4 + T cell specific for E2 and a CTL response to HPV 16 E6 are essential for viral clearance [8]. The cell-mediated immune response is closely followed by production of antibodies to the major capsid protein, L1 [9]. The vast majority of these antibodies are IgG class [10]. After a natural HPV16 infection, 50–70% of the infected individuals produce anti-capsid protein L1 antibodies against HPV16 in cervicovaginal secretions (CVS) [11]. Significant amounts of capsid specific IgG antibody transudated in CVS are sufficient to protect against HPV infection [12, 13]. There are two types of neutralizing L1 antibodies. One hinders cell surface binding while the other prevents binding to the basement membrane [14]. Both seem to prevent the viral internalization either by directly binding or blocking necessary conformational changes [15, 16]. Nevertheless, the exact mechanism(s) by which antibody protects against HPV infection at cervicovaginal mucosal sites is uncertain.

The anti-L1 antibody can directly induce a neutralizing of the infectious virions extracellularly, preventing HPV16 virus from entering the cell [17, 18]. For viruses are characterized by non-entry neutralizing epitopes, some antibody opsonized viruses infect the host cells in different entry patterns, and once inside the cytosol, could be degraded by cytosolic tripartite motif containing-21 (TRIM21)-mediated antibody-dependent intracellular neutralization (ADIN) [19, 20].

TRIM21 consisting of three conserved functional domains, is the only known cytosolic IgG receptor in mammals [21]. It has been demonstrated that once inside the host cell, IgG-coated viruses are bound by the Fc receptor TRIM21 via its carboxy(C)-terminal PRYSPRY domain, which targets virions for segregating and unfolding via ATPase p97/valosin-containing protein (VCP) and degradation by E3Ub-proteasome pathway and also initiates a signaling cascade activating NF-κB, AP-1, and IRF transcription factors and promotes the production of cytokines [22–24]. This intracellular neutralization depending on the widely expressed cytosolic Fc binding protein TRIM21 plays a critical role in eliminating viruses from the infected cells and local anti-viral immune response [25].

The data from the Human Protein Atlas reveal that the cervicovaginal squamous epithelial cells actually express TRIM21 [26]. For HPV16 virus, if the endosome becomes damaged or the fusion leads to the opening of the endosomal compartment to cytosol, then TRIM21 could be effective. It has been implicated that some HPV16 particles with an intact capsid protein L1 structure could escape leaky endosomal compartment into the cytosol before virus entry into the nucleus, and the accompanying L1 protein retains conformation-dependent epitopes, which are recognized and opsonized by anti-L1 antibody [27–29]. Once entering the cytosol, anti-L1 opsonized HPV16 virus can be detected by Fc receptor TRIM21, triggering ADIN for HPV16 virus.

In this study, we aim to explore whether anti-L1 antibody-bound HPV PsV could enter the cytosol and trigger TRIM21-mediated degradation of HPV16 PsV in HEK 293FT cells expressing TRIM21 via proteasomal pathway. Our results indicate that anti-L1 antibody-bound HPV16 PsV in HEK 293FT cells could be degraded intracellularly by TRIM21-mediated activation of the proteasomal pathway.

## Materials and methods

### Antibodies and reagents

Anti-HPV16 L1 antibody [CamVir 1] (ab69), which reacts with a 56 kD protein in cells infected with L1-vaccinia virus, and also reacts very strongly with biopsy specimens containing HPV-16 virion [15], and APC/Cy7® Conjugation Kit (ab102859) and mouse IgG1 [APC/Cy7] (ab46739) as an isotype control were purchased from Abcam plc. (Cambridge, MA, USA). MG132(S2619), which effectively blocks the proteolytic activity of the 26S proteasome complex; Carbobenzoxy-L-leucyl-L-leucyl-L-leucinal (CAS:13340-82-6), MF C26H41N3O5, MW(g/mol) 475.62 and a selective, potent and reversible ATP-competitive p97 inhibitor DBeq (S7199), 2,4-Quinazolinediamine, N2, N4-bis(phenylmethyl)-(CAS:177355-84-9) MF C22H20N4, MW(g/mol)340.42 were purchased from Selleck Chemicals, USA; Brij58(C104448), a polyoxyethylene 20 cetyl ether (CAS:9004-95-9), MF C16H33(OCH2CH2)20-OH, MW(g/mol)1122 was purchased from Sangon Biotech, Shanghai, China; TransEasy transfection regent in Opti-MEM (TEO-01011) was purchased from FOREGENE CO.LTD., Chengdu, China; Plasmid-Safe ATP-Dependent DNase(E3101k) was purchased from Epicentre Biotech; Benzonase Nuclease(E1014) was purchased from Sigma.

### Plasmids

p16sheLL plasmid encoding HPV16 L1 and L2 (Plasmid #37320, Addgene) was kindly donated by Dr. John Schiller (NIH, Bethesda USA); pRL-SV40-C (D2768), pNFκB-TA-Luc (D2207), pAP1-TA-Luc plasmids (D2109) and Dual Luciferase Reporter Gene Assay Kit (RG027) were purchased from Beyotime Biotechnology, Shanghai, China.

### Primers for real-time qPCR

All primers used in this study were designed (Table 1) using Primer Express 3.0 software (Applied Biosystems) and Primer-BLAST (NCBI), and synthesized by Tsingke Biotech Co. Ltd, China. Thermal cycling procedures for real-time qPCR were performed routinely.

**Table 1:** Primer sequences for qPCR assays

Primers	Sequence	Used for
P1	F: 5′-CGAGTGACAAGCCTGTAGCCC-3′	TNF-α
P2	R: 5′-CCTTGAAGAGGACCTGGGAGT-3′	
P3	F: 5′-GAGCAAGGACCCTCACGACC-3′	IRF-3
P4	R: 5′-GGCCAACACCATGTTACCCAGT-3′	
P5	F: 5′-ACCAGTCCATCCCAGTGGCT-3′	IRF5
P6	R: 5′-TTCGGTGTATTTCCCTGTCTCC-3′	
P7	F: 5′-TACCTAGAGTACCTCCAGAACAG-3′	IL-6
P8	R: 5′-CATTTGCCGAAGAGCCCTCA-3′	
P9	F: 5′-GCAGCTGAGAATCCTGGG-3′	TRIM21
P10	R: 5′-CAGCCCTGGCACATGGC-3′	
P11	F: 5′-TCGTGACCACCCTGACCTACGGCGTG-3′	eGFP
P12	R: 5′-CACCTTGATGCCGTTCTTCTGCTTGTCG-3′	

### Cell line and culture

HEK-293FT cell line was obtained from Invitrogen™ (#R70007). All cells were maintained in Dulbecco’s modified Eagle’s medium (DMEM) supplemented with 10% fetal bovine serum, 2 mmol/L L-glutamine, 1 mmol/L sodium pyruvate, and 100U/mL penicillin and 40U/mL gentamicin at 37 °C, 5% CO_2_.

### Preparation of HPV16 PsV

HPV16 PsV were generated in HEK-293FT cells, as described previously [30]. Briefly, 7.5 × 10^6^ cells of HEK-293FT were planted in a 75-cm^2^ flask overnight and co-transfected with p16Shell and plasmids pcDNA3.1-eGFP by using Trans Easy transfection reagent (FOREGENE), then harvested, washed and resuspended with DPBS-Mg; The cell suspension obtained was lysed and matured with addition of 10% Brij-58 (final concentration of ~ 0.35%), Benzonase and Plasmid Safe(final concentration of ~ 0.25% each) for 24 h at 37℃, and NaCl was added to a final concentration of 850 mmol/L to achieve a high titer HPV16 PsV lysate stock; the clarified lysate was purified as follows: gently laid the clarified cell lysate onto the top of step gradient of Optiprep Density Gradient Medium (Sigma D1556)(39, 33, 27% w/v) and spun at 234,000×*g* at 16℃ for 3.5 h. After centrifugation, fractions were collected from the top layer and selected the one demonstrating the highest titration of infectivity for further experiments.

For PsVs titer detection [30], HEK-293FT cells were seeded at 2 × 10^5^ cell/well in 12-well flat-bottom plate and incubated, and 500 μL of PsV (lysate stock 1:250 to 1:8000 dilution with serum-free DMEM) and 500 μL of DMEM containing 10% FSC were added the following day. After 48 h, the cells were harvested, suspended in the loading buffer, and subjected to flow cytometry (FCM) with GFP-FITC (BD FACS Canto II). PsV titer was calculated as following formula: [2 μL/mL] × [stock dilution] × [cells at time of infection] × [fraction of positive cells].

### Western blot for L1 of HPV16 PsV

10 μL HPV16 PsV lysate stock was boiled with 10 μL 5 × SDS-PAGE loading buffer (Solarbio) for 5 min, and centrifuged at 12,400×*g*, 4 °C for 10 min, the supernatant was collected and electrophoresed in 10% SDS-PAGE gel and transferred electrophoretically onto a PVDF membrane. The blocked membrane with 5% skim milk in PBST was incubated with anti-HPV16 L1 antibody [CamVir 1,ab69] (Abcam) at a dilution of 1:1000, 4℃ overnight and then with HRP-conjugated goat anti-mouse IgG for 45 min at room temperature and then washed with PBST 4 times for 5 min each time. The result was revealed with chemiluminescence substrate and ImageQuant LAS500.

### Detection of anti-L1 antibody-bound HPV16 PsV

HEK-293FT cells were plated at 2 × 10^5^cells/well in 12-well flat-bottom plate and cultured overnight. 500 μL of HPV16 PsV (1:2000) or anti-HPV16 L1 antibody-bound HPV16 PsV (two-fold serial dilution of anti-L1 antibody) were added to the plate and incubated for 4 h; the cells were refreshed with a culture medium for another 48 h incubation, and then were washed 2 times with phosphate-buffered saline (PBS). DNA was extracted for detection of HPV16 PsV copy number by using qPCR as described above; Furthermore, HPV16 PsV (lysate stock 1:2000 dilution) were pre-incubated with APC/Cy7 labeled anti-L1 antibody (ab69) (1.25 μg) for 1 h and added to HEK-293FT cells seeded at 2 × 10^5^ cells/well in 12-well flat-bottom plate for 4 h incubation, then the culture medium was refreshed and cultured for an additional 24 h. The cells were harvested, suspended in the loading Buffer and subjected to FCM with GFP-FITC and APC-Cy7 for detection of anti-L1 antibody-bound HPV16 PsV.

### qPCR and RT-qPCR

DNA was extracted by using a TIANamp Genomic DNA Kit (TIANGENE) according to the manufacturer’s protocol. A total of 100 ng of DNA as a template was used to detect the copy number of PsV, Log dilution of pcDNA3.1-eGFP plasmid was used as a standard curve; Total RNA was isolated by using TRIzol™ Reagent (Invitrogen) according to the manufacturer’s protocol. 1 μg RNA was reverse-transcribed into cDNA by using Eastep RT Master Mix Kit (Promega) at 20 μL reaction volume, and 1 μL cDNA was used for qPCR.

### Detection for TRIM21 expression

HEK-293FT cells were plated at 2 × 10^5^cells/well in 12-well flat-bottom plate and cultured overnight. 500 μL of HPV16 PsV (1:2000) or anti-HPV16 L1 antibody-bound HPV16 PsV (1:400 dilution) were added to the plate and incubated for 4 h, the cells were refreshed with a culture medium for another 48 h incubation, and then were washed 2 times with PBS. Total RNA was extracted for the detection of expression of TRIM21 by using RT-qPCR as described above.

### Detection for HPV16 PsV load

HEK-293FT cells were plated at 2 × 10^5^cells/well in 12-well flat-bottom plate and cultured overnight. 500 μL of HPV16 PsV (1:2000) or anti-HPV16 L1 antibody-bound HPV16 PsV (anti-HPV16 L1 antibody 1:400 dilution) were added to the plate and incubated for 4 h. In addition, cells were pretreated with 10 μmmol/L MG132 or 10 μmmol/L DBeq for 4 h, 500 μL of HPV16 PsV (1:2000) or anti-HPV16 L1 antibody-bound HPV16 PsV(1:400 dilution) were added to the plate and incubated for 4 h; the cells were refreshed with culture medium for another 48 h incubation, and then were washed 2 times with phosphate-buffered saline(PBS). DNA was extracted for detection of the copy number of PsV by using qPCR as described above.

### Detection of transcription factors

HEK-293FT cells were planted in 12-well flat-bottom plate overnight, and co-transfected with and without 1 μg pRL-SV40-C plus 1 μg pNFκB-TA-Luc or 1 μg pAP1-TA-Luc for 4 h; the cells were refreshed with culture medium supplemented with or without 10 μM MG132 or 10 μM DBeq for 4 h; the cells were infected with HPV16 PsV (1:2000 dilution) and anti-L1 IgG-bound HPV16 PsV (anti-L1 antibody 1.25 μg) for 4 h, and refreshed with 500 μL of culture medium for another 48 h incubation. The activity of NF-κB, AP-1 and mRNA expression of interferon regulatory factors 3 and 5 (IRF3, IRF5) were determined by using Dual-luciferase reporter assay and qPCR assay, respectively.

### Detection for expression of cytokines

HEK-293FT cells were plated at 2 × 10^5^cells/well in 12-well flat-bottom plate and cultured overnight. Cells were pretreated with or without 10 μM DBeq or MG132 for 4 h, 500 μL of HPV16 PsV (1:2000) or anti-HPV16 L1 antibody-bound HPV16 PsV (anti-L1 antibody 1.25 μg) were added to the plate and incubated for 4 h, the cells were refreshed with culture medium for another 48 h incubation, and then were washed 2 times with PBS. Total RNA was extracted for detection of expression of interleukin-6 (IL-6), tumor necrosis factor α (TNFα) and interferon α (IFNα).

### Statistical analysis

Data were summarized using descriptive statistics of mean and standard deviation. ANOVA was used as a statistical test with SPSS 17.0 software, and P-value, less than 0.05 was considered significant.

## Results

### Characterization of HPV16 PsV

**Fig. 1:**
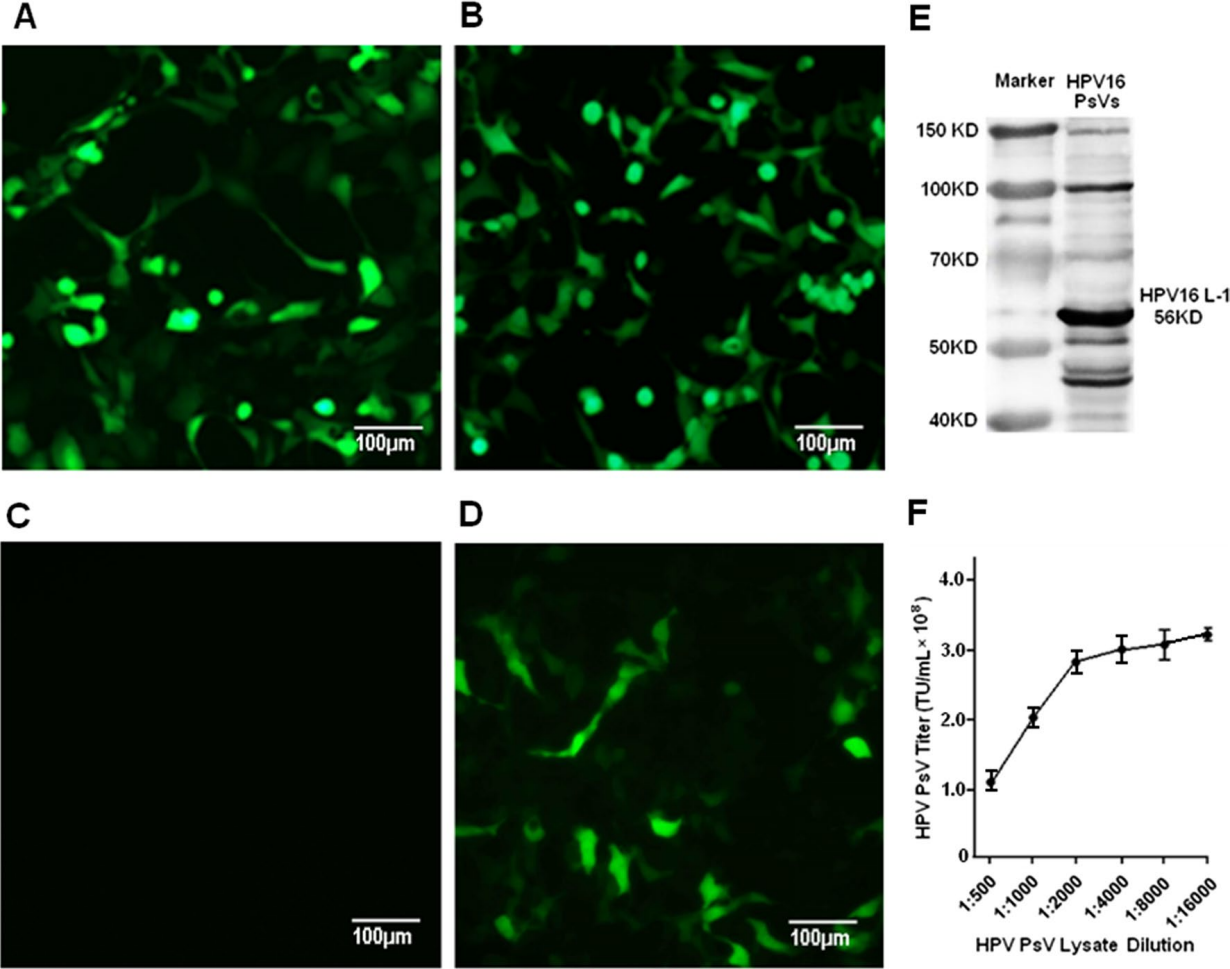
Production and characterization of HPV16 PsV. **A** and **B** represent the microscopic fluorescent images of HEK-293FT cells transfected with pcDNA-eGFP and pcDNA-eGFP/p16Shell, respectively; **C** and **D** represent the microscopic fluorescent images of HEK-293FT cells infected with the HPV16 PsV lysate stock from HEK-293FT cells transfected with pcDNA-eGFP and pcDNA-eGFP/p16Shell, respectively; **E** represents L1 protein expression of HPV16 PsV detected by WB; **F** represents the titer of HPV16 PsV of the lysate stock in FCM assay

HPV16 PsV generated from HEK-293FT cells co-transfected with p16Shell and plasmids pcDNA3.1-eGFP (GFP +) was shown to be able to infect HEK-293FT cells efficiently in a short time of HPV16 PsV exposure (Fig. 1A–D) as documented by microscopic fluorescent imaging. Western blotting showed that the L1 major capsid protein (56KD) was expressed at a high level and self-assembled into HPV16 PsV particles (Fig. 1E). In addition, HPV16 PsV contained a high titer of 3.35 × 10^8^ TU/mL at 1:16,000 dilution of the lysate stock as determined by FCM assay (Fig. 1F).

### Anti-L1 antibody-bound HPV16 PsV shows the ability to infect cells

**Fig. 2:**
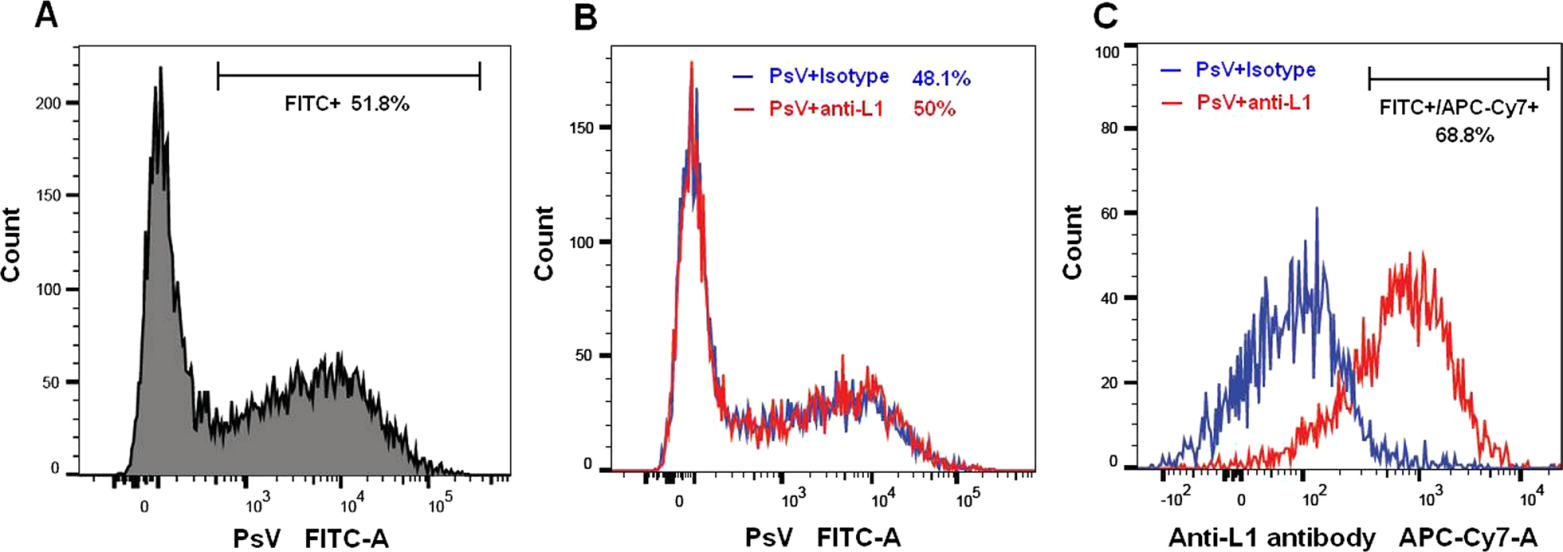
FCM analysis for HPV16 PsV competence to infect cells. **A** represents the rate of FITC^+^ cells infected with HPV16 PsV; **B** and **C** represent the percentages of FITC^+^ and FITC^+^/APC-Cy7^+^ cells infected with anti-L1 bound HPV16 PsV and with HPV16 PsV plus anti-L1 isotype, respectively

Based on that antibodies can routinely enter cells attached to viral particles and mediated an intracellular immune response [31], we propose that anti-L1-bound HPV16 PsV particle would retain its competence to infect cells. FCM assay showed that the cells with FITC^+^/APC-Cy7^+^ HPV16 PsV accounted for 68.8% of the FITC^+^ cells following exposure to anti-L1-bound HPV16 PsV tagged with FITC/APC-Cy7 for 24 h(Fig. 2A–C), suggesting that anti-L1 antibody could engage with HPV16 PsV to form an anti-L1-bound HPV16 PsV particle that infects cells effectively.

### HPV16 PsV-bound anti-L1 antibody up-regulates TRIM21 expression and reduces PsV load

**Fig. 3:**
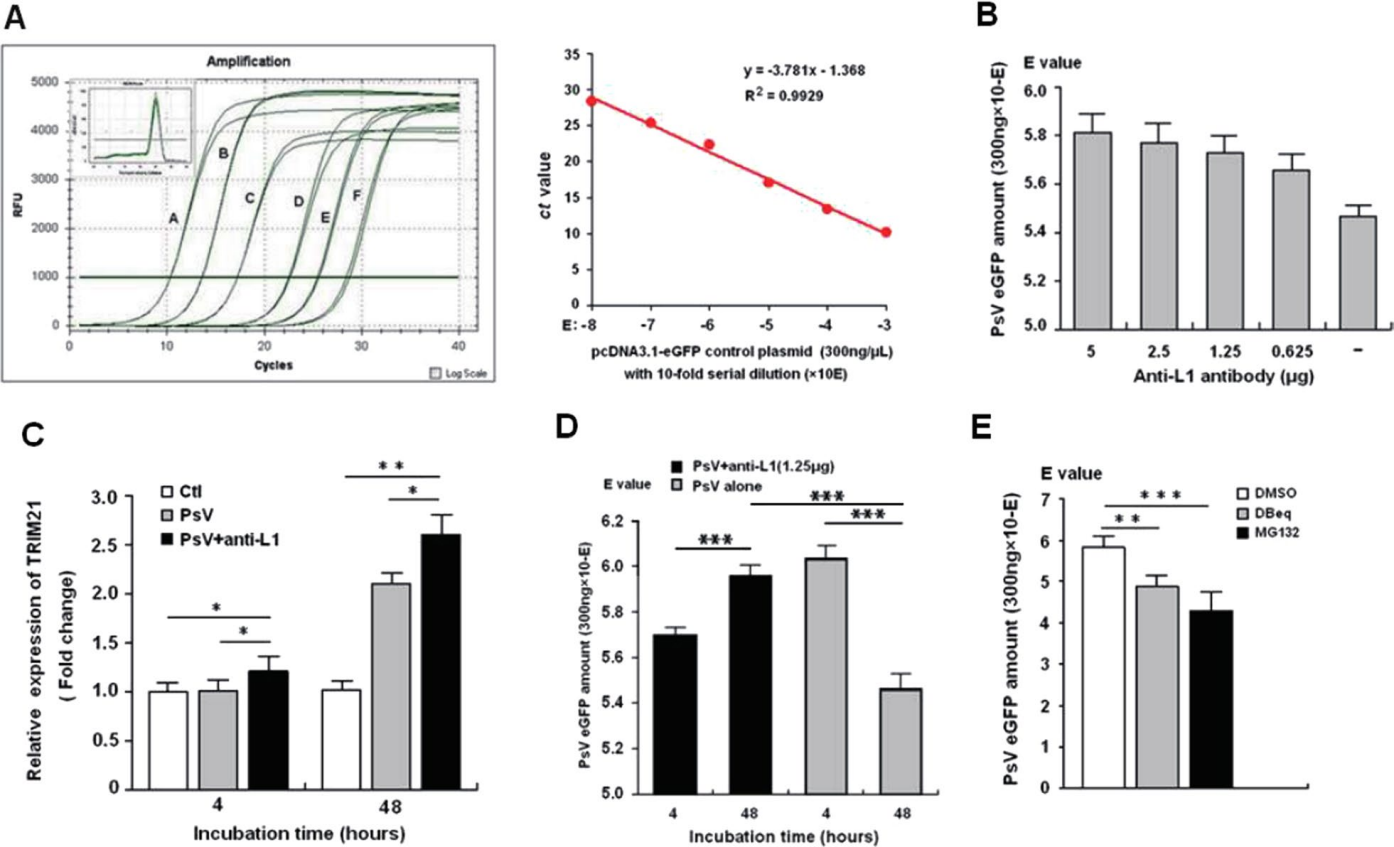
Determination for HPV16 PsV load of the infected cells by qPCR assay. **A** represents the specificity and sensitivity of qPCR assay; **B** represents an optimal amount of anti-L1 antibody to bind to HPV16 PsV; **C** represents the expression level of TRIM21 trigged by HPV16 PsV-bound anti-L1 antibody; **D** represents the anti-L1 antibody-mediated degradation of HPV16 PsV; **E** represents the impact on the degradation of anti-L1-bound HPV16 PsV by inhibiting VCP and proteasome activity. All experiments were performed in triplicate, and data are expressed as mean ± SD (n = 3). Error bars represent the SD of replicating data points. *P < 0.05; **< 0.01; ***< 0.001, compared to the control

Using a specific qPCR assay (Fig. 3A), our results showed that the expression level of TRIM21 mRNA was significantly increased following exposure of HEK-293FT cells to HPV16 PsV coated with an optimal amount of anti-L1 antibody (1.25 μg) (Fig. 3B) and to HPV16 PsV alone for 4 h and 48 h, respectively, compared to that of the control cells (P < 0.05 and P < 0.001) (Fig. 3C). Furthermore, HPV16 PsV load in the cells infected with anti-L1-bound HPV16 PsV was significantly decreased after 48 h, compared to that of the 4 h; while compared to 4 h after incubation, the viral load of the cells incubated with PsV alone was dramatically higher after 48 h (P < 0.001); and also after 48 h of infection, viral load in the PsV + anti-L1 group was significantly lower than that in the PsV group (Fig. 3D). However, by inhibiting proteasome activity, HPV16 PsV load in the infected cells was apparently increased, compared to the vehicle control (P < 0.001). These results suggest that HPV16 PsV-bound anti-L1 antibody would be degraded intracellularly.

### HPV16 PsV-bound anti-L1 antibody up-regulates the expression of transcription factor

**Fig. 4:**
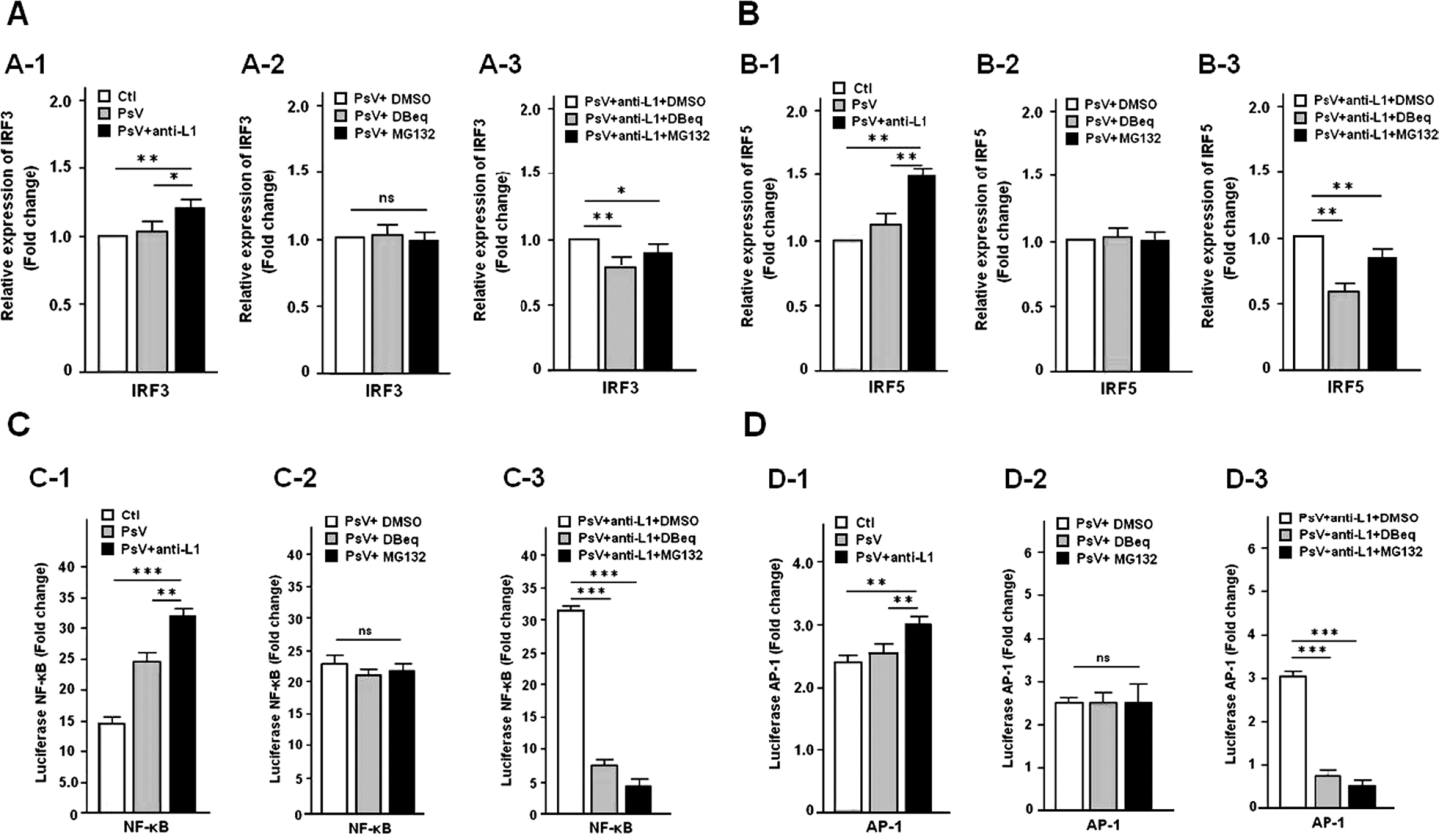
Determination for expression of transcription factors by qPCR and luciferase assays. **A** and **B** represent the expression level of IRF3 and IRF5 by qPCR assay, respectively; **C** and **D** represent the expression level of NF-κB and AP-1 by luciferase assay, respectively. All experiments were performed in triplicate, and data are expressed as mean ± SD (n = 3). Error bars represent the SD of replicate data points. *P < 0.05; **< 0.01; ***< 0.001, ns: no significance, compared to the control

It is essential in ubiquitin–proteasome mediated expression of transcription factors for TRIM21-mediated intracellular a virus degradation response upon recognition of antibody [23, 32, 33], we examined whether anti-L1 antibody induces the expression of IFR3, IRF5, NF-κB and AP-1 by HPV16 PsV infected cells pretreated with or without VCP inhibitor DBeq and proteasome inhibitor MG132. qPCR and luciferase reporter assays revealed that the relative mRNA expression (fold-change) of IFR3/IRF5, NF-κB and AP-1 were significantly increased in the infected cells but decreased in those treated with the two inhibitors, compared to the corresponding controls (Fig. 4A–D). The results showed that ubiquitins released from TRIM21 recruited proteasome promote the expression of these transcription factors.

### HPV16 PsV-bound anti-L1 antibody up-regulates the expression of proinflammatory cytokines

**Fig. 5:**
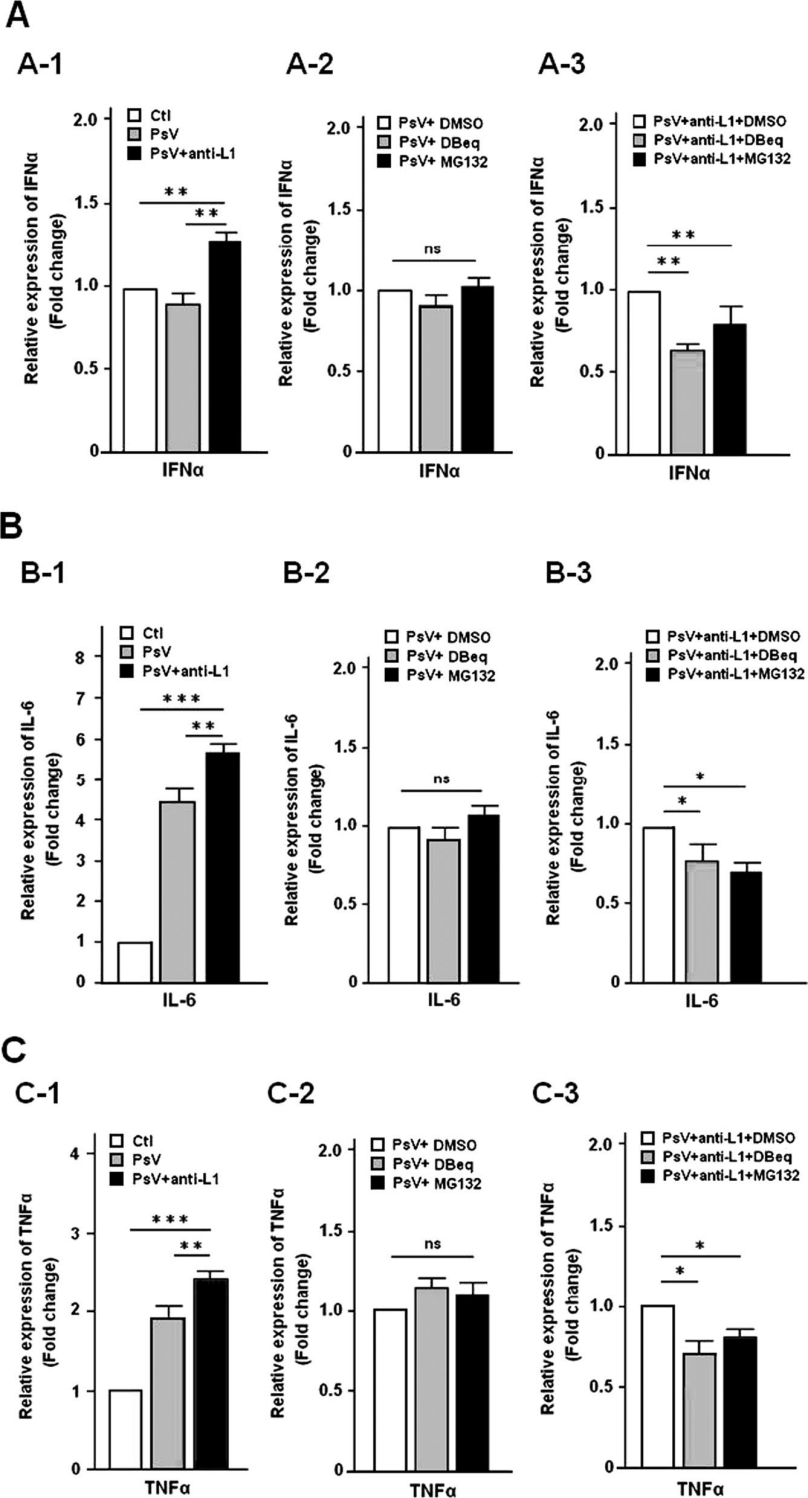
Determination for expression of proinflammatory cytokines by qPCR. **A** represents the expression level of IFNα; **B** represents the expression level of IL-6 and **C** represents the expression level of TNFα. All experiments were performed in triplicate, and data are expressed as mean ± SD (n = 3). Error bars represent the SD of replicate data points. *P < 0.05; **< 0.01; ***< 0.001, ns: no significance, compared to the control

Based on that proinflammatory cytokines play an important role in TRIM21 signaling-mediated intracellular response of virus infection [23, 34], we determined whether anti-L1 antibody induces expression of IFNα, IL-6 and TNFα by HPV16 PsV infected cells pretreated with or without VCP inhibitor DBeq and proteasome inhibitor MG132. qPCR assay showed that the relative mRNA expression (fold-change) of IFNα, IL-6 and TNFα was significantly up-regulated in the infected cells but decreased in the ones pretreated with the two inhibitors, compared to the corresponding controls (Fig. 5A–C). The results suggest that the anti-L1 antibody facilitates the expression of proinflammatory cytokines by HPV16 PsV infected cells for the reduction in virus load due to possible neutralization.

## Discussion

Cervicovaginal mucosal immunity is required for the prevention and clearance of hrHPV infection. Anti-L1 antibody predominates in cervicovaginal secretions of natural hrHPV-infected or -vaccinated women and might play a critical role in the immunologic defense of the cervicovaginal mucosa against hrHPV infection [9, 35–37]. In addition to the traditional barrier function of IgG antibody to hrHPV in neutralizing hrHPV infection in the extracellular environment, several studies have revealed that antibody-mediated protection extends to the cytosolic compartment of cells. This postentry viral defense mechanism requires the binding of the antibody to a cytosolic Fc receptor TRIM21 [17, 19].

Cytosolic IgG–virion complexes colocalize with the high-affinity IgG receptor TRIM21, which recognizes an IgG Fc epitope via its C-terminal PRYSPRY domain. After binding a virion-associated antibody, TRIM21 targets the cytosolic IgG–virus complex for proteasomal degradation in an antibody-dependent intracellular neutralization (ADIN) [38]. In addition to mediating ADIN, TRIM21 synthesizes lysine-63 polyubiquitin chains and activates the NF-κB, AP-1, and IRF3/IRF5/IRF7 immune signaling pathways, and drives the production of proinflammatory cytokines such as IFNα, TNFα and IL-6 to induce an anti-viral state in infected cells, whereas viral clearance in the absence of antibody or TRIM21 is significantly reduced [23].By recruiting TRIM21, IgG mediates virus neutralization in the cytosol and activates a second or last line of immune defense against invading viruses, and TRIM21 provides important protection against viral infection [31].

In this study, we found that 34.4% of HEK-293FT cells could be infected by anti-L1 antibody-bound HPV16 PsV (Figs. 1, 2). Importantly, we demonstrated that anti-L1 antibody-bound HPV16 PsV were capable of eliciting the up-regulation of TRIM21 expression by HEK 293FT cells and, therefore may represent one protective response of TRIM21 against intracellular infection of HPV16 PsV. Furthermore, we showed that HPV16 PsV loads were significantly reduced in the presence of anti-L1 antibody (Fig. 3D) (P < 0.001), illustrating that anti-L1 antibody could trigger TRIM21-mediated ADIN of HPV16 PsV in the cytosol. Moreover, we showed that HPV16 PsV-bound anti-L1 antibody remarkably increased TRIM21-involved signaling cascade responses of proteasome pathway by which both of expression of transcription factors NF-κB, AP-1, and IRF3/IRF5 and proinflammatory factors IFNα, IL-6 and TNFα were significantly up-regulated (Figs. 4, 5) (P < 0.01). Conversely, we also found that inhibition of proteasomal activity increased HPV16 PsV load (Fig. 3E) and down-regulated the expression of transcription and proinflammatory factors (Figs. 4, 5). Thus, these findings suggest that anti-L1 antibody-bound HPV16 PsV may leak from the endocytic vesicle into the cytosol and could be detected by TRIM21, as proposed by several studies [23, 27, 29]. As it enters cells along with HPV16 virus, anti-L1 antibody-bound HPV16 PsV appears able to interact with intracellular TRIM21, resulting in ubiquitin proteasomal pathway mediated degradation of HPV16 PsV.

The role for anti-L1 antibody proposed here would have a particularly important role in defending against intracellular HPV16 infection. As suggested by our results, a possible explanation is that intracellular anti-L1-specific antibody could trigger TRIM21-mediated degradation of HPV16 virus and promotes virus clearance in cervicovaginal squamous epithelial cells. As opposed to cell-mediated cytotoxicity, anti-L1 antibody mediated intracellular HPV16 virus neutralization might not destroy the infected cells [19]. Therefore, the levels of anti-L1 antibodies in CVS of natural HPV16 infected women and TRIM21 expression by the cervicovaginal squamous epithelial cells could play a critical role in neutralizing and clearing of persistent HPV16 virus infection.

## Conclusion

In conclusion, our results suggest that anti-L1 antibody-induced TRIM21 mediated ADIN of HPV16 PsV may represent a potential mechanism for reducing persistent HPV16 infection but is required for further validation in the clinical setting.

## Data Availability

The data and material generated or analyzed in this study are available upon reasonable request, and could be provided by JM Huang (hjianming@yahoo.com).

## Abbreviations

PsV: Pseudovirus
HrHPV: High-risk human papillomavirus
CVS: Cervicovaginal secretions
TRIM21: Cytosolic tripartite motif containing-21
ADIN: Antibody-dependent intracellular neutralization
VCP: Valosin-containing protein
TF: Transcription factors
NF-κB: Nuclear factor kappa-B
AP-1: Activator protein 1
IRF: Interferon regulatory factor
IFNα: Interferon α
IL6: Interleukin 6
TNFα: Tumor necrosis factor α
